# Population and Component Community Descriptors of Monopisthocotyla Parasitizing *Triportheus* spp. (Actinopterygii: Triportheidae) in the Tocantins-Araguaia River Basin, Brazil

**DOI:** 10.1007/s11686-026-01372-4

**Published:** 2026-09-07

**Authors:** Dennisiane de Jesus Saraiva, Andréa Pereira da Costa, Diego Carvalho Viana, Rita de Maria Seabra Nogueira, Simone Chinicz Cohen

**Affiliations:** 1 Laboratory of Fish Helminth Parasites, Oswaldo Cruz Institute, Oswaldo Cruz Foundation – Fiocruz, Rio de Janeiro, RJ Brazil; 2 Postgraduate Program in Biodiversity and Health, Oswaldo Cruz Institute, Oswaldo Cruz Foundation – Fiocruz, Rio de Janeiro, RJ Brazil; 3 Postgraduate Program in Animal Science, State University of Maranhão – UEMA, São Luís, MA Brazil; 4 Center for Advanced Morphophysiological Studies, State University of the Tocantina Region of Maranhão – UEMASUL, Imperatriz, MA Brazil

**Keywords:** Dactylogyridae, Branchial parasites, Parasite ecology, Community structure, *Triportheus *

## Abstract

**Purpose:**

Monopisthocotyla constitute a diverse group of ectoparasites of Neotropical fish and are recognized as important bioindicators of environmental conditions in aquatic ecosystems. Despite the high biodiversity of the Tocantins–Araguaia basin, studies simultaneously describing the population descriptors and component communities of these parasites in fish of the genus *Triportheus* are still scarce. This study aimed to characterize the population and component community descriptors of Monopisthocotyla parasitizing *Triportheus albus* and *Triportheus trifurcatus* in the Tocantins River, Maranhão, Brazil.

**Methods:**

A total of 262 hosts were examined (69 *T. albus* and 193 *T. trifurcatus*). Parasites were collected from the gills, morphologically identified, and evaluated for prevalence, mean intensity, mean abundance, and distribution pattern. Component communities were characterized using species richness, Shannon diversity, Pielou’s evenness, Simpson’s diversity and dominance, and Berger–Parker dominance.

**Results:**

*Triportheus trifurcatus* showed higher richness, diversity, and evenness, while *T. albus* displayed higher dominance, mainly due to the high proportional abundance of *Jainus* spp. Most parasite populations showed an aggregated distribution pattern, and host-related factors exerted limited influence on parasite abundance and richness. The findings demonstrate differences in the organization of component communities between the two host species.

**Conclusions:**

The combined use of population and component community descriptors improved the understanding of the ecological organization of Monopisthocotyla communities parasitizing Neotropical fish. These findings provide baseline information for future biodiversity and environmental monitoring studies in the Tocantins-Araguaia River basin.

## Introduction

The Neotropical region harbors one of the greatest diversities of freshwater fish in the world, especially in South America, which is home to more than 5,000 described species and represents an important center of ichthyofaunal biodiversity [1, 2]. In this context, the order Characiformes represents one of the most diverse groups, comprising more than 2,000 described and valid species, whose diversification is associated with paleogeographic, hydrogeological, climatic, and biogeographic factors characteristic of this region [3–8].

Brazil stands out in this ecoregion, especially in large hydrographic systems such as the Tocantins–Araguaia basin, the second largest water system contained entirely within this country. With its high species richness, this basin is under increasing anthropogenic pressure, mainly due to the construction of hydroelectric plants and changes in the hydrological regime [9, 10]. Despite its relevance, knowledge about the parasitic fauna in this basin is still fragmented in comparison to that of the Amazon system, although recent studies indicate high diversity of Monopisthocotylea endemic to the region [11, 12].

Standing out among these parasites are Monopisthocotyla, with their direct life cycle, high specificity, and close association with their hosts, which makes them significant elements in the structuring of Neotropical parasitic communities [13–17]. Currently, they are recognized as a paraphyletic group, and recently Brabec et al. [18] proposed elevating the subclasses Monopisthocotyla and Polyopisthocotyla to class status, supplanting the class Monogenea or Monogenoidea.

Monopisthocotyla act as sensitive biological indicators of environmental conditions [19–21]. The structure of their communities is influenced by a combination of biotic factors, such as body size and trophic niche of the hosts, and abiotic factors that include temperature and seasonality [22–24].

In environments impacted by dams and changes in hydrological flow, such as the Tocantins River, it is important to monitor these branchial ectoparasites, since their abundance and prevalence may reveal environmental changes and physiological conditions of the hosts [25, 26]. These factors directly influence quantitative descriptors, such as prevalence, mean intensity, and mean abundance, as well as the parasite distribution pattern, which is often characterized by aggregation [22, 27].

Among the most representative hosts of South American ichthyofauna are species of the genus *Triportheus* (Characiformes: Triportheidae), which are fundamental to trophic dynamics and the regional economy [28, 29]. However, knowledge about the parasitic fauna of *Triportheus* in the Tocantins–Araguaia basin is still limited, especially with respect to component community descriptors and ecological patterns of monogeneans, highlighting a significant gap in the understanding of parasite-host interactions in river systems impacted by dams [15, 16, 30].

Given this scenario, this study aimed to characterize the population and community descriptors of Monopisthocotyla parasitizing *Triportheus albus* (Cope, 1872) and *Triportheus trifurcatus* (Castelnau, 1855) in the Tocantins–Araguaia basin. Specifically, the prevalence, mean intensity, mean abundance, and distribution pattern of the parasite populations were evaluated, as well as the richness, diversity, evenness, and dominance of the component communities. The possible influence of host sex, body size, seasonality, and sampling sites on parasite abundance and richness was also investigated.

## Materials and Methods

Specimens of the fish genus *Triportheus* were collected at two points along the Tocantins River, in the municipality of Imperatriz, state of Maranhão, Brazil. The two sampling points were located relatively close to each other in a section of the river under anthropogenic influence. Point 1 (5°32′34.598′′ S, 47°29′20.801′′ W) corresponded to an area close to the river’s edge, characterized by less influence from the current, predominantly sandy substrate, and the presence of domestic effluent discharges in the proximities. Point 2 (5°32′43.071′′ S, 47°29′16.583′′ W) was located closer to the main channel of the river, showing greater influence from the water flow and sandy substrate.

Samples were collected in August 2019, October 2020, August and September 2022, and July 2024, and were classified as originating from the dry season. Samples collected in December 2021, April and May 2022, and June and December 2023 were categorized as coming from the rainy season. This seasonal classification was used as a categorical variable in the analyses of infestation descriptors.

The fish were caught using a casting net, placed in plastic bags, and taken to the Laboratory of Ecology and Limnology (LEL) of the State University of the Tocantina Region of Maranhão (UEMASUL), where they were identified, cataloged, and processed for the Monopisthocotylea collection. Voucher specimens of the hosts were deposited in the Ichthyological Collection of the Center for Agricultural and Environmental Sciences (CICCAA) at the Federal University of Maranhão (UFMA), under accession numbers: CICCAA 02472 and CICCAA 02473 (*Triportheus trifurcatus*), and CICCAA 02474, CICCAA 02475, and CICCAA 02476 (*Triportheus albus*).

The hosts were necropsied, and their gills were removed and placed in heated distilled water (~ 65 °C) to promote relaxation and detachment of the parasites. Absolute ethanol was then added to reach a final concentration of 70%, and the material was fixed. The gill filaments were examined under a stereomicroscope at the Laboratory of Parasitology and Parasitic Diseases (LPDP) of the State University of Maranhão (UEMA), and the parasites were collected for further processing and identification.

For morphological analysis using light microscopy, the specimens were mounted in Hoyer’s medium for observation of sclerotized structures and subsequent taxonomic identification. Some of the specimens were kept at the generic level (*Anacanthorus* spp., *Ancistrohaptor* spp., and *Jainus* spp.) due to the need for complementary taxonomic analyses for specific delimitation.

The quantitative descriptors of prevalence (P), mean intensity (MI), and mean abundance (MA) were calculated for each species, as outlined by Bush et al. [31]. The distribution pattern of each parasite population was evaluated using the Dispersion Index (DI), with values greater than 1 considered indicative of an aggregation tendency. The significance of this pattern was verified by the *d* statistic, where values greater than 1.96 were interpreted as statistically significant evidence of an aggregated distribution.

To characterize the Monopisthocotyla component communities associated with each host species, the following ecological community descriptors were calculated: species richness (*S*), corresponding to the number of recorded species; Shannon–Wiener diversity index (*H’*), using natural logarithm; Pielou’s evenness index (*J’*), to evaluate the uniformity of abundance distribution among species; Simpson’s diversity index (1-D); Simpson’s dominance index (*D*); and Berger–Parker dominance index (*d*), used to estimate the proportion of individuals belonging to the most abundant species in the component community. All indices were calculated based on the total abundance of each parasite species recorded across the pool of individuals of each host species, representing the Monopisthocotyla component communities [32, 33].

Differences in prevalence among host species and between seasonal periods were assessed using the Chi-square test (x²) with Yates’s correction. The influence of sex and seasonal period (dry vs. rainy) on parasite abundance and richness was evaluated using the Mann-Whitney U test. The influence of standard length (cm) and total weight (g) of the hosts on species abundance and richness was determined using Spearman’s rank correlation coefficient (r_s_). All the statistical analyses were performed using SPSS version 32 software, adopting a significance level of 5% (p < 0.05). Community descriptors were calculated using R software version 4.6.1, with the ‘dplyr’ and ‘vegan’ packages.

The community indices were used descriptively to characterize the organization of the component communities associated with each host species and were not subjected to comparative statistical analyses.

To calculate the component community descriptors, all recorded taxa were considered, including those identified only at the genus level (*Anacanthorus* spp., *Ancistrohaptor* spp., and *Jainus* spp.), as they represented distinct taxonomic units within the parasite community.

For the analyses of species composition and species sharing among hosts, only taxa identified to the species level were considered. Records identified only to the genus level (*Anacanthorus* spp., *Ancistrohaptor* spp., and *Jainus* spp.) were excluded from these analyses to avoid overestimating species richness.

Specimen collection was authorized by the Chico Mendes Institute for Biodiversity Conservation (ICMBio), under Permit no. 69,275. All the procedures involving animals were performed according to the standards of the National Council for the Control of Animal Experimentation (CONCEA) and were approved by the Ethics Committee on Animal Use (CEUA) at the State University of Maranhão (UEMA), under Protocol no. 09/2019.

## Results

Two hundred and sixty-two specimens of fish of the genus *Triportheus* were examined, comprising 69 individuals of *Triportheus albus* (Cope, 1872) and 193 individuals of *Triportheus trifurcatus* (Castelnau, 1855) for biometric data (Table 1).

**Table 1:** Biometric characteristics of *Triportheus albus* and *Triportheus trifurcatus* collected in the Tocantins–Araguaia basin, Maranhão, Brazil

Host	*N*	SL (cm)	*R* (cm)	TW (g)	*R* (g)
*Triportheus albus*	69	14.3 ± 1.2	10.9–17.2	41.2 ± 10.1	20.0–74.0
*Triportheus trifurcatus*	193	14.8 ± 1.4	11.0–18.2	49.7 ± 13.8	18.0–84.0

N = number of examined hosts; SL = standard length; R = range; TW = total weight. Standard length and total weight values are expressed as mean ± standard deviation, followed by the minimum and maximum observed range

Among the total number of hosts examined, 49 individuals of *T. albus* (71.01%) and 147 individuals of *T. trifurcatus* (76.17%) were parasitized by Monopisthocotyla. General infection descriptors revealed values of mean intensity of 8.04 and mean abundance of 5.71 in *T. albus*, and mean intensity of 7.05 and mean abundance of 5.37 in *T. trifurcatus*. The composition of parasite fauna included representatives of the genera *Anacanthorus*, *Ancistrohaptor*, and *Jainus*. Nine species of Monopisthocotyla were recorded in *T. albus* and 13 species in *T. trifurcatus*. In *T. albus*, the species recorded were *Anacanthorus alatus* Kritsky, Boeger & Van Every, 1992, *Anacanthorus chaunophallus* Kritsky, Boeger & Van Every, 1992, *Anacanthorus chelophorus* Kritsky, Boeger & Van Every, 1992, *Anacanthorus cornutus* Kritsky, Boeger & Van Every, 1992, *Anacanthorus lygophallus* Kritsky, Boeger & Van Every, 1992, *Anacanthorus quinqueramus* Kritsky, Boeger & Van Every, 1992, *Anacanthorus* spp., *Ancistrohaptor* spp. and *Jainus* spp. The following parasites were identified in *T. trifurcatus*: *Anacanthorus acuminatus* Kritsky, Boeger & Van Every, 1992, *Anacanthorus andersoni* Kritsky, Boeger & Van Every, 1992, *Anacanthorus chaunophallus* Kritsky, Boeger & Van Every, 1992, *Anacanthorus chelophorus* Kritsky, Boeger & Van Every, 1992, *Anacanthorus cornutus* Kritsky, Boeger & Van Every, 1992, *Anacanthorus euryphallus* Kritsky, Boeger & Van Every, 1992, *Anacanthorus formosus* Kritsky, Boeger & Van Every, 1992, *Anacanthorus lygophallus* Kritsky, Boeger & Van Every, 1992, *Anacanthorus* spp., *Ancistrohaptor falcatum* Agarwal & Kritsky, 1998, *Ancistrohaptor falciferum* Agarwal & Kritsky, 1998, and *Ancistrohaptor* spp. and *Jainus* spp. The parasitic fauna was characterized by species belonging to the genera *Jainus*, *Anacanthorus*, and *Ancistrohaptor*. *Jainus* spp. showed the highest prevalence and mean abundance values in both hosts, with a prevalence of 57.14%, mean intensity of 6.1, and mean abundance of 3.49 in *T. albus*, and a prevalence of 62.89%, mean intensity of 3.63, and mean abundance of 2.28 in *T. trifurcatus* (Table 2).

**Table 2:** Ecological descriptors and distribution pattern of Monopisthocotyla parasitizing *Triportheus albus* and *Triportheus trifurcatus* collected in the Tocantins–Araguaia basin, Maranhão, Brazil

Hosts	*Triportheus albus*						*Triportheus trifurcatus*					
Monopisthocotyla	*P* (%)	MI	MA	DI	d	Distribution	*P* (%)	MI	MA	DI	d	Distribution
*Anacanthorus alatus*	5.71	1	0.06	0.94	-0.49	Random	—	—	—	—	—	—
*Anacanthorus acuminatus*	—	—	—	—	—	—	2.58	1.4	0.04	1.79	7.91	Aggregated
*Anacanthorus andersoni*	—	—	—	—	—	—	0.52	1	0.01	1	0	Random
*Anacanthorus chaunophallus*	10	1.57	0.16	1.58	3.38	Aggregated	26.29	2.12	0.56	3.27	9.55	Aggregated
*Anacanthorus chelophorus*	18.57	1.38	0.26	1.29	1.73	Aggregated	24.23	2.19	0.53	3.41	10.04	Aggregated
*Anacanthorus cornutus*	4.29	1	0.04	0.96	-0.24	Random	3.61	1.14	0.04	1.19	1.98	Aggregated
*Anacanthorus euryphallus*	—	—	—	—	—	—	1.55	1.33	0.02	1.46	4.59	Aggregated
*Anacanthorus formosus*	—	—	—	—	—	—	0.52	1	0.01	1	0	Random
*Anacanthorus lygophallus*	7.14	1.8	0.13	2.41	8.19	Aggregated	5.15	1.4	0.07	1.62	6.11	Aggregated
*Anacanthorus quinqueramus*	4.29	1.33	0.06	1.43	2.49	Aggregated	—	—	—	—	—	—
*Anacanthorus* spp.	38.57	3	1.16	3.21	13.82	Aggregated	44.85	2.28	1.02	2.94	8.41	Aggregated
*Ancistrohaptor falcatum*	—	—	—	—	—	—	3.61	1.14	0.04	1.19	1.98	Aggregated
*Ancistrohaptor falciferum*	—	—	—	—	—	—	2.58	1	0.03	0.97	-0.18	Random
*Ancistrohaptor* spp.	5.71	1.75	0.1	2.03	5.97	Aggregated	28.87	2.43	0.7	3.86	11.48	Aggregated
*Jainus* spp.	57.14	6.1	3.49	8.91	23.36	Aggregated	62.89	3.63	2.28	4.65	22.71	Aggregated
**TOTAL**	**71.01**	**8.04**	**5.71**	—	—	—	**76.17**	**7.05**	**5.37**	—	—	—

*P* = prevalence (%); MI = mean intensity; MA = mean abundance; DI = dispersion index; d = *d* statistic. Values of DI > 1 indicate a tendency toward aggregation, whereas d > 1.96 denotes statistically significant aggregation. The “TOTAL” row represents the overall infection descriptors for each host species. A dash (—) indicates that the parasite species was not recorded in the respective host

The analysis of community descriptors revealed differences in the organization of Monopisthocotyla component communities associated with the two hosts. The component community of *T. trifurcatus* showed higher taxon richness (*S* = 13), higher Shannon–Wiener diversity (*H’* = 1.642), higher Pielou’s evenness (*J’* = 0.640), and higher Simpson’s diversity (1-D = 0.743). In *T. albus*, lower richness (*S* = 9), lower Shannon–Wiener diversity (*H’* = 1.187), lower evenness (*J’* = 0.540), and lower Simpson’s diversity (1-D = 0.557) were observed, corresponding to a higher Simpson’s dominance (*D* = 0.443). These results indicate a higher concentration of abundance in a few taxa within the *T. albus* component community, mainly *Jainus* spp. (Table 3). Furthermore, the Berger–Parker dominance index was higher in *Triportheus albus* (0.622) than in *T. trifurcatus* (0.427), indicating a higher numerical dominance of a single Monopisthocotyla species in the *T. albus* component community.

**Table 3:** Community descriptors of Monopisthocotyla component communities associated with *Triportheus albus* and *Triportheus trifurcatus* from the Tocantins–Araguaia basin, Maranhão, Brazil

Hosts	S	H’	J’	1-D	D	d
*Triportheus albus*	9	1.187	0.540	0.557	0.443	0.622
*Triportheus trifurcatus*	13	1.642	0.640	0.743	0.257	0.427

S = Species Richness; H’ = Shannon-Wiener Diversity Index; J’ = Pielou’s Evenness Index; 1-D = Simpson’s Diversity Index; D = Simpson’s Dominance Index; d = Berger-Parker Dominance Index

**Fig. 1:**
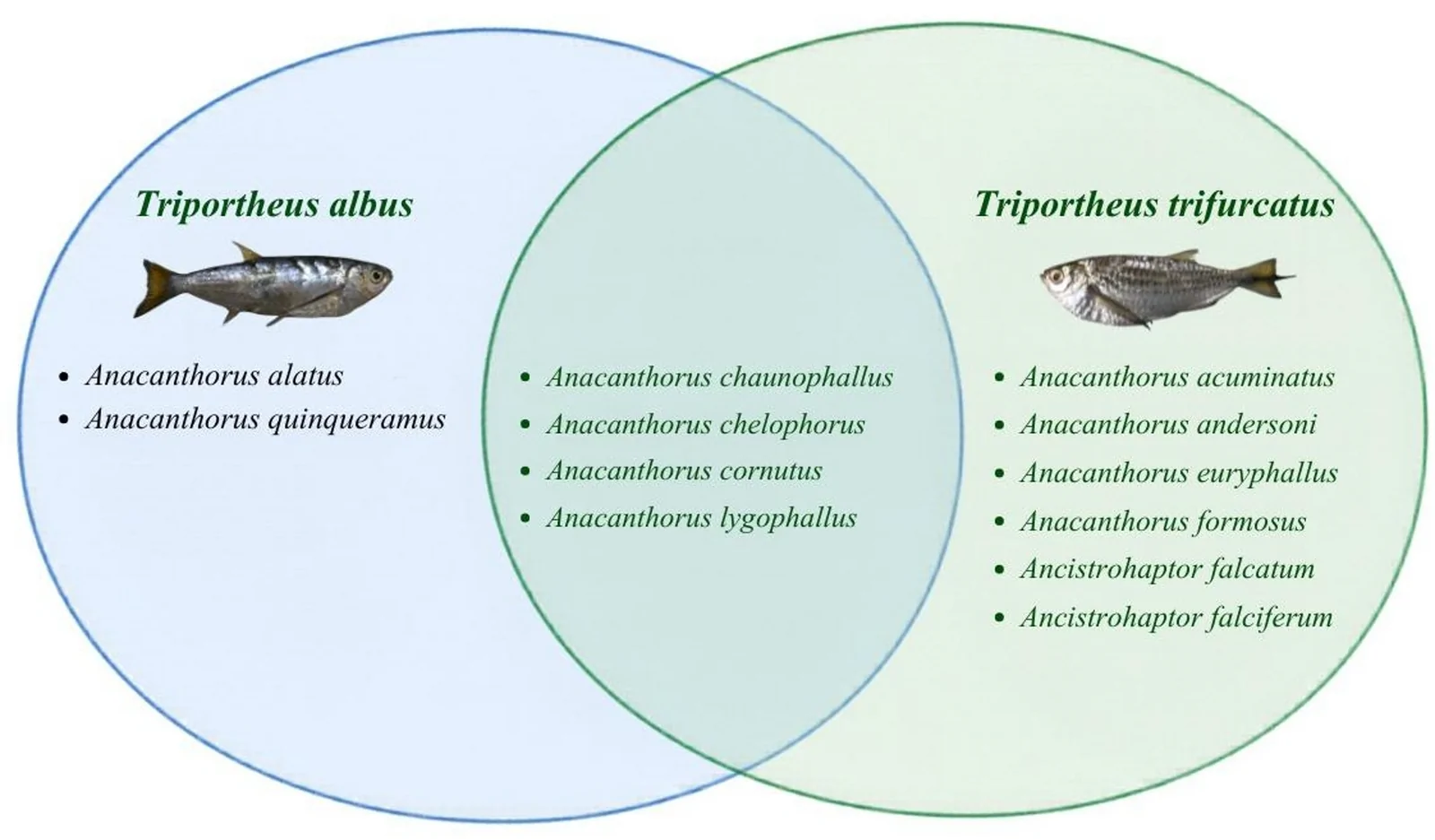
Venn diagram representing the composition of Monopisthocotyla species recorded on *T. albus* and *T. trifurcatus* in the Tocantins–Araguaia basin, Maranhão, Brazil, highlighting species shared between the hosts and species exclusive to each host

Of the total number of recorded species, four were shared between the hosts, while two occurred exclusively on *T. albus* and six exclusively on *T. trifurcatus* (Table 4; Fig. 1).

**Table 4:** Composition of the Monopisthocotyla fauna recorded in *Triportheus albus* and *Triportheus trifurcatus* collected in the Tocantins–Araguaia basin, Maranhão, Brazil

Monopisthocotyla	*Triportheus albus*	*Triportheus trifurcatus*
*Anacanthorus alatus*	+	−
*Anacanthorus acuminatus*	−	+
*Anacanthorus andersoni*	−	+
*Anacanthorus chaunophallus*	+	+
*Anacanthorus chelophorus*	+	+
*Anacanthorus cornutus*	+	+
*Anacanthorus euryphallus*	−	+
*Anacanthorus formosus*	−	+
*Anacanthorus lygophallus*	+	+
*Anacanthorus quinqueramus*	+	−
*Ancistrohaptor falcatum*	−	+
*Ancistrohaptor falciferum*	−	+

(+) species recorded in the host; (–) species not recorded in the host

Among the shared species, *Anacanthorus chaunophallus* and *Ancistrohaptor* spp. showed significant differences in prevalence between hosts, with higher values of both found in *T. trifurcatus* (*A. chaunophallus*: χ² = 7.039; *p* = 0.008; *Ancistrohaptor* spp.: χ² = 14.448; *p* < 0.001). Although the general descriptors of infestation were similar between hosts, *T. trifurcatus* showed higher parasite richness than *T. albus*. In addition, some species were found to infest only one host. For example, *Anacanthorus alatus* and *A. quinqueramus* occurred solely in *T. albus*, while *A. acuminatus*, *A. andersoni*, *A. euryphallus*, *A. formosus*, *Ancistrohaptor falcatum* and *A. falciferum* were found exclusively in *T. trifurcatus*.

An analysis of the distribution pattern of parasitic populations revealed a predominance of aggregated distribution in both host species. Seven of the nine recorded species showed an aggregated pattern *T. albus*, while nine of the 13 recorded species showed this pattern in *T. trifurcatus*. *Jainus* spp. showed the highest Dispersion Index (DI) values (*T. albus*: DI = 8.91; d = 23.36; *T. trifurcatus*: DI = 4.65; d = 22.72), followed by *Anacanthorus* spp. and *Ancistrohaptor* spp., indicating strong aggregation of these parasite populations.

The influence of the hydrological regime on the ecological descriptors was evaluated considering the dry and rainy seasons. Sixty-six individuals of *T. albus* and 68 of *T. trifurcatus* were analyzed during the dry season, and three individuals of *T. albus* and 125 of *T. trifurcatus* in the rainy season (Table 5).

**Table 5:** Biometric and sampling characteristics of *T. albus* and *T. trifurcatus* collected in the Tocantins–Araguaia basin, Maranhão, Brazil

Host	Season	*N*	Males	Females	Mean TL (cm)
*Triportheus albus*	Dry	66	20	46	14.67
*Triportheus albus*	Rainy	3	3	0	16.47
*Triportheus trifurcatus*	Dry	68	45	23	17.05
*Triportheus trifurcatus*	Rainy	125	54	71	18.27

N = number of examined hosts; Mean TL = mean total length of the hosts (cm)

The prevalence of Monopisthocotyla in *T. albus* was 71.21% in the dry season and 100% in the rainy season, while its mean abundance was 5.88 in the dry season and 8.00 in the rainy season. Despite the differences observed in the descriptive values, there was no significant difference in parasite abundance between the periods (U = 67.0, *p* = 0.419). On the other hand, the prevalence of Monopisthocotyla in *T. trifurcatus* was 72.06% in the dry season and 78.40% in the rainy season, while its mean abundance was 9.56 in the dry season and 11.30 in the rainy season. Notwithstanding the higher descriptive values in the rainy season, no significant difference in parasite abundance was detected between the seasonal periods (U = 3905.0, *p* = 0.284).

As for sex distribution, 23 males and 46 females of *T. albus* and 99 males and 94 females of *T. trifurcatus* were examined (Table 5). An analysis of the influence of host sex on parasite abundance and richness did not reveal significant differences (*T. albus*: U = 503.0, *p* = 0.742 for abundance and U = 504.0, *p* = 0.748 for richness; *T. trifurcatus*: U = 4502.0, *p* = 0.695 for abundance and U = 4553.5, *p* = 0.795 for richness). Moreover, no significant biometric correlations were found between standard length or total weight and parasite abundance or richness in *T. albus* (*p* > 0.05). In *T. trifurcatus*, a significant, albeit tenuous, positive correlation was observed between the hosts’ standard length and Monopisthocotyla richness (*r*_*s*_ = 0.148, *p* = 0.039), indicating a tendency of increasing parasite species richness in hosts of greater length (Table 6).

**Table 6:** Influence of host sex on the abundance and richness of Monopisthocotyla in *T. albus* and *T. trifurcatus* collected in the Tocantins–Araguaia basin, Maranhão, Brazil

Host	Variable	U	*p*
*Triportheus albus*	Abundance	503.0	0.742
	Richness	504.0	0.748
*Triportheus trifurcatus*	Abundance	4502.0	0.695
	Richness	4553.5	0.795

U = Mann-Whitney U test statistic; *p* = value of significance

Regarding sampling points, 44 individuals of *T. albus* were examined at point 1 and 25 at point 2. Monopisthocotyla showed a prevalence of 70.45% at point 1 and of 72.00% at point 2, with mean abundances of 5.75 and 5.64, respectively. No significant differences in parasite abundance were detected between the points (U = 528.5, *p* = 0.790). Ninety-two *T. trifurcatus* individuals were examined at point 1 and 101 at point 2. Monopisthocotyla prevalence was 78.26% at point 1 and 74.26% at point 2, with mean abundances of 5.65 and 5.12, respectively. Similarly, no significant differences in parasite abundance were observed between the sampling points (U = 3989.0, *p* = 0.087) (Table 7).

**Table 7:** Influence of sampling points on the ecological descriptors of Monopisthocotyla in *T. albus* and *T. trifurcatus* collected in the Tocantins–Araguaia basin, Maranhão, Brazil

Host	Point	*N*	*P* (%)	MA	U	*p*
*Triportheus albus*	Point 1	44	70.45	5.75	528.5	0.790
	Point 2	25	72.00	5.64	—	—
*Triportheus trifurcatus*	Point 1	92	78.26	5.65	3989.0	0.087
	Point 2	101	74.26	5.12	—	—

N = number of examined hosts; *P* = prevalence (%); MA = mean abundance; U = Mann-Whitney U test statistic; *p* = value of significance

## Discussion

The population and community descriptors of Monopisthocotyla parasitizing *Triportheus* spp. in the Tocantins-Araguaia basin revealed different aspects of the ecological organization of these component communities. On the whole, the parasite populations evaluated in this study were characterized by high prevalence and a predominance of aggregated distribution, while the component community descriptors revealed differences in richness, diversity, evenness, and dominance between the two host species. These patterns are consistent with those reported for parasite communities of Neotropical systems in different Brazilian river basins [15, 34, 35].

Although the hosts share part of the taxonomic composition, the community indices revealed differences in the organization of the component communities. The higher Shannon–Wiener and Simpson diversity in *T. trifurcatus* indicates a more balanced distribution of abundances among taxa. In contrast, the higher Simpson’s dominance observed in *T. albus* demonstrates that a significant portion of the parasite individuals was concentrated in a few taxa, mainly *Jainus* spp. Differences in host susceptibility, availability of gill microhabitats, and parasite transmission success may contribute to this pattern [15, 24, 27].

This pattern was also corroborated by the Berger–Parker index, which revealed higher dominance of a single parasite species in *T. albus*. This result indicates that a high proportion of individuals in the component community belonged to *Jainus* spp., whereas the lower values observed in *T. trifurcatus* reflect a more balanced distribution of proportional abundances among Monopisthocotyla taxa. These findings demonstrate that the organization of component communities depends not only on richness and total abundance but also on the relative distribution of individuals among taxa. Thus, communities with similar abundance values may exhibit different levels of diversity, evenness, and dominance.

The predominance of Dactylogyridae, particularly that of species of the genera *Anacanthorus* and *Ancistrohaptor*, reinforces the parasite-host association frequently observed in Monopisthocotyla of *Triportheidae* fish in Neotropical river systems [34, 36]. This predominance suggests that species of the genus *Triportheus* maintain relatively conserved parasite infracommunity structures across different hydrographic systems, although species composition and relative abundance may vary depending on local environmental characteristics. While Monopisthocotyla have been identified as the predominant parasites in species such as *Triportheus rotundatus* in the Amazon [15], other studies show that different species may prevail depending on the environment. For example, in the Amazon River system, the ciliate *Ichthyophthirius multifiliis* was the predominant species in *T. angulatus* and *T. curtus*, although Monopisthocotyla such as *Anacanthorus pitophallus* were still significant components of the community [37]. This variation suggests that, although Monopisthocotyla are characteristic of *Triportheus*, their predominance may be influenced by environmental factors and by ectoparasite community composition [37].

The high prevalence (> 70%), abundance, and species richness values observed in this study are consistent with the patterns described for Monopisthocotyla in Neotropical fish, where infestation levels often vary significantly according to host species and geographical locations [15]. In some tributaries of the Amazon, *T. rotundatus* showed 100% prevalence of metazoan infestation, with Monopisthocotyla being the main culprit [34]. In the Tocantins-Araguaia basin, the structure of these communities is likely influenced by host behavior and aggregation, and by the availability and survival of oncomiracidia in the environment, which is similar to patterns observed in other rivers in northern Brazil [34, 37]. Overall, these findings reinforce the idea that Monopisthocotyla communities in *Triportheus* spp. exhibit high structural stability on a regional scale.

The aggregated distribution pattern observed for most parasite populations constitutes one of the most consistent population results of this study, as has been observed in *Triportheus* species from the Amazon and in other cichlids in the Paraná River [34, 37, 38]. This pattern, whereby most parasites are concentrated in a few host individuals, is generally attributed to differences in host susceptibility or to localized environmental conditions that favor transmission [34, 38].

Furthermore, the positive correlation between host length and parasite component-community richness in *T. trifurcatus* (*r*_*s*_=0.148) suggests that larger fish offer a greater gill surface area for colonization and that their accumulated exposure time has been longer. Tavares-Dias et al. [15] and Borges et al. [35], discuss a similar pattern and state that the body length of host fish showed a positive, albeit weak, correlation with the prevalence, intensity, and abundance of Monopisthocotylea species.

This weak intensity of association may indicate that, for pelagic schooling fish such as those of the genus *Triportheus*, population density and social behavior exert a more significant influence on transmission dynamics than the individual size of the host [34, 39]. As these authors observed, the high rate of contact among individuals in gregarious species facilitates the encounter of Monopisthocotyla with the gills, making the structure of the parasite component community more dependent on school cohesion than on fish biometrics [34, 39].

The absence of significant differences in parasite abundance between sexes and hydrological periods suggests relative stability of the Monopisthocotyla component communities associated with *Triportheus* spp. in the Tocantins River. Similar patterns have been observed in other Neotropical Characiformes, in which parasite transmission can remain relatively constant even in the face of seasonal variations in the hydrological regime [34, 37]. However, these results should be interpreted cautiously, since the absence of statistical significance does not necessarily imply the absence of ecological effects [40]. This pattern suggests that the hydrological regulation imposed by the cascade of dams in the Tocantins basin may be stabilizing parasite-host interactions by reducing natural peak flows, which, in free-flowing rivers, would act to dilute infective forms and to disperse hosts [40]. Studies of impacted rivers indicate that anthropogenic changes in the flow regime can modify the structure of parasitic communities, making them less dependent on natural seasonal variations and more influenced by the environment’s artificial stability [41].

This scenario favors the maintenance of constant levels of infestation and continuous reproduction of Monopisthocotyla throughout the year [14–16]. Furthermore, the similarity of male and female infection levels confirms that the two sexes exploit similar environments and have the same susceptibility, a pattern frequently observed in other Characiformes from the Amazon and Tocantins basins [16, 35].

The Tocantins-Araguaia basin has been less exploited than the Amazon and Paraná basins. Ecological data for Monopisthocotyla in *Triportheus* spp. also serve as important environmental indicators [15, 16]. Given that Monopisthocotyla are sensitive to water quality, the high prevalence and stability of the communities observed here may reflect chronic stress of the system or eutrophication processes resulting from the slowing down of the stretch under study [16, 25, 30].

In this context, the Monopisthocotyla identified here act as sentinels of ecosystem health, since the dynamics of their populations respond rapidly and measurably to local anthropogenic pressures. Thus, the patterns observed in this study contribute to heighten our understanding about the ecology of Monopisthocotyla in neotropical systems under anthropogenic influence, expanding the body of knowledge about the organization of parasitic communities in under-explored river basins.

Despite the relevance of the results obtained, some limitations must be considered when interpreting the findings of this study. Sampling was conducted at two sites within the same stretch of the Tocantins–Araguaia basin, which warrants caution when extrapolating the observed patterns to other locations in the basin or to different sampling periods. Furthermore, some Monopisthocotyla taxa were identified only to the genus level. Although these taxa were retained in the calculations of the component community descriptors, since they represented distinct taxonomic units in the samples, the lack of specific identification precluded their use in analyses of species composition and host-species sharing.

Another important limitation concerns the asymmetry in sampling effort across hydrological periods for *Triportheus albus*, as only three individuals were collected during the rainy season. This reduced sample size decreases the statistical power of the seasonal analyses and may have limited the detection of temporal differences in ectoparasite abundance. Therefore, the absence of statistically significant seasonal differences should be interpreted with caution. Future studies with broader spatial and temporal scope, combined with deeper taxonomic resolution and the integration of molecular tools, could expand the understanding of the factors that structure Monopisthocotyla component communities in *Triportheus* species.

## Conclusions

This study characterized the population and component community descriptors of Monopisthocotyla associated with *Triportheus albus* and *Triportheus trifurcatus* in the Tocantins–Araguaia basin. The parasite populations showed a predominance of an aggregated distribution, whereas the component communities differed in richness, diversity, evenness, and dominance. *Triportheus trifurcatus* presented a more diverse component community with a more uniform distribution of abundances, while *T. albus* displayed higher dominance, mainly of *Jainus* spp. The results reinforce the importance of jointly using population and community descriptors to understand different levels of the ecological organization of Neotropical fish parasites. Furthermore, they provide baseline information for future monitoring studies in areas under anthropogenic influence.

## Data Availability

No datasets were generated or analysed during the current study.
